# Recent Advances on Surface-Modified GBM Targeted Nanoparticles: Targeting Strategies and Surface Characterization

**DOI:** 10.3390/ijms24032496

**Published:** 2023-01-27

**Authors:** Francesca Rodà, Riccardo Caraffi, Silvia Picciolini, Giovanni Tosi, Maria Angela Vandelli, Barbara Ruozi, Marzia Bedoni, Ilaria Ottonelli, Jason Thomas Duskey

**Affiliations:** 1 Clinical and Experimental Medicine, University of Modena and Reggio Emilia, 41125 Modena, Italy; 2 IRCCS Fondazione Don Carlo Gnocchi ONLUS, 20148 Milan, Italy; 3 Nanotech Lab, TE.FAR.T.I., Department of Life Sciences, University of Modena and Reggio Emilia, 41125 Modena, Italy

**Keywords:** nanomedicine, glioblastoma, surface characterization, targeted nanoparticles, anticancer nanomedicine

## Abstract

Glioblastoma multiforme (GBM) is the most common malignant brain tumor, associated with low long-term survival. Nanoparticles (NPs) developed against GBM are a promising strategy to improve current therapies, by enhancing the brain delivery of active molecules and reducing off-target effects. In particular, NPs hold high potential for the targeted delivery of chemotherapeutics both across the blood–brain barrier (BBB) and specifically to GBM cell receptors, pathways, or the tumor microenvironment (TME). In this review, the most recent strategies to deliver drugs to GBM are explored. The main focus is on how surface functionalizations are essential for BBB crossing and for tumor specific targeting. We give a critical analysis of the various ligand-based approaches that have been used to target specific cancer cell receptors and the TME, or to interfere with the signaling pathways of GBM. Despite the increasing application of NPs in the clinical setting, new methods for ligand and surface characterization are needed to optimize the synthesis, as well as to predict their in vivo behavior. An expert opinion is given on the future of this research and what is still missing to create and characterize a functional NP system for improved GBM targeting.

## 1. Introduction

Brain tumors are a wide and heterogenous class of neoplasms that differentiate in epidemiology, prognosis, and histological, molecular, and clinic characteristics [1,2]. Among them, glioblastoma multiforme (GBM) is the most aggressive and common primary brain tumor. GBM is mainly diagnosed in males older than 60 years, and the incidence rate varies according to geographical area [3]. Despite the low percentage of new cases each year and advances in molecular profiling and in chemotherapy, GBM represents a major clinical issue in both the medical and the economical setting. Unfortunately, the standard of care and current treatments rarely lead to the successful eradication of the tumor, resulting in a median overall survival of less than 2 years from diagnosis [4,5,6]. The failure of GBM treatment is linked to several factors [7,8,9]. First, GBM has an infiltrative nature and is characterized by intra- and inter-tumor heterogeneity. Moreover, the blood–brain barrier (BBB) is considered one of the main obstacles for the delivery of chemotherapeutics to GBM; due to its protective nature, it prevents the delivery of drugs to the tumor site, minimizing the concentration of therapeutics and decreasing their efficacy. In addition, efflux transporters coupled with GBM stem cells can induce resistance to drugs, such as temozolomide, which is considered the gold standard of chemotherapeutic therapies against GBM. All these factors lead to a poor prognosis and tumor recurrence. Therefore, advanced therapeutic strategies have been proposed and studied to improve patient outcomes. Nanoparticles (NPs) are nanometric systems that have led to groundbreaking discoveries across the board from electronics to imaging, art, etc. In this framework, the application of NPs in the medical field, known as nanomedicine, has opened new opportunities for the treatment of hard-to-treat diseases, e.g., neurodegenerative or genetic diseases, especially GBM [10,11]. Several NPs have been tested to encapsulate traditional drugs in order to enhance drug solubility, encapsulation, and protection in the biological environment, reduce off-target toxicity, and enable passage through the BBB and/or the specific delivery of drugs to GBM via targeting, as well as controlled release at the target site [12,13,14,15,16,17,18,19,20,21,22,23]. The possibility of engineering the surface of NPs with molecules that can promote specific accumulation in the brain or in GBM cells has opened the way toward highly effective treatment options. Unfortunately, the formulation of surface-modified NPs is often lacking a thorough characterization of their surface composition, primarily due to the technical difficulty or high cost of the techniques necessary for this aim, leading to a poor possibility of optimizing the formulation of these NPs.

**Figure 1:**
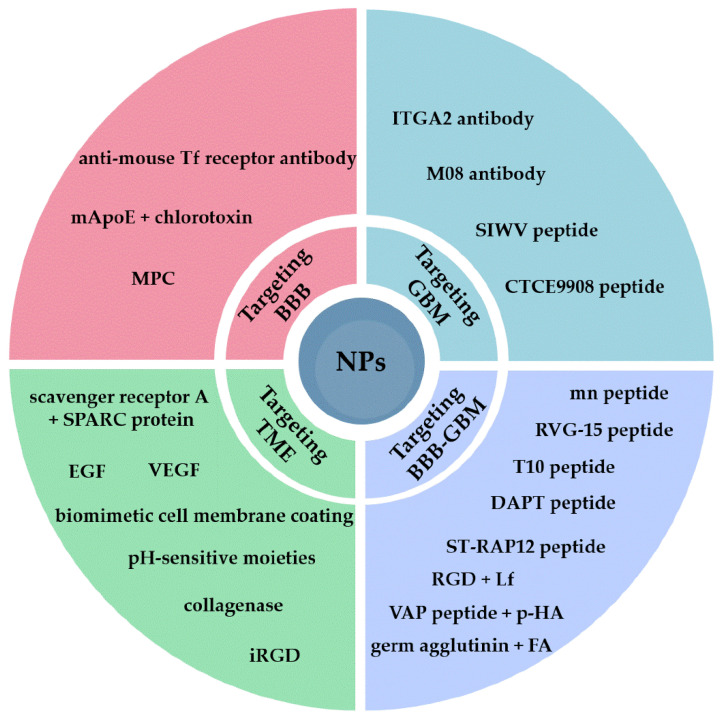
Schematic representation of novel molecules investigated as targeting moieties for the development of targeted NPs, as well as those targeting the TME for GBM therapy. Abbreviations: BBB—blood-brain barrier; EGF—epidermal growth factor; FA—folic acid; GBM—glioblastoma multiforme; Lf—lactoferrin; MPC—2-methacryloyloxyethyl phosphorylcholine; NPs—nanoparticles; p-HA—p-hydroxybenzoic acid; Tf—transferrin; TME—tumor microenvironment; VEGF—vascular endothelial growth factor.

In this review, we highlight the recent research performed to revolutionize NP targeting to improve brain penetration, by specifically targeting the BBB, GBM cells, or the tumor microenvironment (TME) (summarized in Figure 1). Moreover, we look at current methods used to characterize the presence and quantity of targeting moieties on the NP surface, including an expert opinion on where we are and what still needs to be improved to increase our capacity to combat such a frightening and deadly disease.

## 2. Targeting the BBB

The BBB is a strictly selective cellular barrier located between the blood compartment and the brain. On the one hand, it regulates the permeability of small molecules and ions to ensure brain nutrition and an appropriate neuronal function; on the other hand, it prevents unwanted cells and substances from entering the brain. The presence of this highly selective barrier significantly hinders the delivery of chemotherapeutics to the brain and, thus, the treatment of brain cancer. The organization and functioning of the BBB can be altered under pathological conditions; in the case of brain tumors like GBM, a distinct form of the BBB exists and is known as the blood–brain tumor barrier (BBTB). It is well known that the BBTB of GBM patients is disrupted and leaky, which could facilitate the passage of chemotherapeutics to the brain; however, it must be said that this disruption is generally not able to allow the permeation of significant amounts of drugs [24]. Thus, it is necessary to investigate the mechanisms underlying the passage of solutes across the BBB to create BBB-permeable drug delivery systems.

Over the last decades, numerous strategies to improve the delivery of therapeutics to the brain for treating GBM have been investigated, including the use of BBB and BBTB-targeted NPs. These strategies enabled the crossing of these barriers and the penetration of the drug delivery system inside the brain, where it could release its payload to have a therapeutic effect. All these approaches utilized transport mechanisms such as passive diffusion, which depends on physicochemical properties (e.g., lipophilicity/hydrophilicity, charge, and molecular weight), or active transport, with a carrier-mediated transport or a receptor-mediated transport. In this context, receptors and transporters overexpressed on the BBB, such as transferrin (Tf) [25], insulin [26], low-density lipoprotein [27], lactoferrin (Lf) [28], nicotinic receptors [29], and glucose [30] and choline transporters [31], have been extensively studied and exploited to improve the targeting and crossing of the BBB by NPs, with the aim of GBM treatment; however, the recent literature on BBB-permeable systems evidences how targeting single receptors and transporters has limited efficacy. On the contrary, recent studies tended to focus on the use of dual or multiple ligands to maximize the possibility to cross the BBB. For example, Han and coworkers developed a formulation of nanocapsules consisting of a cleavable crosslinked peptide and 2-methacryloyloxyethyl phosphorylcholine (MPC), a molecule that contains a choline and an acetylcholine analogue. The aim was to enhance the delivery across the BBB or the BBTB of a therapeutic monoclonal antibody via choline transporter- or acetylcholine nicotinic receptor-mediated transport, in order to suppress the tumor proliferation. Results showed that nimotuzumab could be effectively delivered to the central nervous system in orthotopic U87-EGFRwt glioma xenograft mouse models, thanks to the presence of the dual targeting moiety [32]. Formicola et al. designed and developed liposomes composed of cholesterol, sphingomyelin, and DSPE–PEG–maleimide, functionalized with a modified apolipoprotein E-derived peptide (CWG-LRKLRKRLLR; mApoE) and a neurotoxin, chlorotoxin, demonstrating their synergistic effects in improving the permeability of doxorubicin-loaded liposomes across a human cell-based BBB model. Furthermore, they demonstrated that targeted doxorubicin-loaded liposomes were able to reduce the viability of GBM U87 cells seeded in the basolateral compartment to the same extent as free doxorubicin (−76.6%), but without damaging the endothelial monolayer [33]. Notwithstanding these interesting results, this study was limited to in vitro experiments, demonstrating that still much work needs to be conducted to translate those liposomes to a clinic setting. A slightly different approach was investigated by Galstyan and others. Here, the authors developed nano-immunoconjugates (NIC) consisting of a poly(β-l-malic acid) (PMLA) backbone, to which checkpoint inhibitor antibodies were covalently attached, namely, cytotoxic T-lymphocyte-associated antigen 4 (a-CTLA-4) and programmed cell death-1 (PD-1) antibodies. These two antibodies can help activate the local antitumor immune response in the brain; however, their direct administration is unsuccessful due to their inability to cross the BBB. For this reason, the antibodies were covalently linked to the PMLA backbone, which was also modified by conjugation with an anti-mouse Tf receptor antibody to enable BBB crossing using Tf receptor-mediated transport. NIC treatment of mice bearing intracranial GL261 GBM resulted in an increase in CD8^+^ T cells, NK cells, and macrophages with a corresponding decrease of regulatory T cells in the brain tumor area [34]. This study is a successful example of delivery across the BBB of polymer-conjugated antibodies for immunotherapy of brain tumors, which could lead to an effective GBM treatment via activation of the local brain tumor immune response.

## 3. Targeting GBM Cell Receptors

Active targeting to promote BBB crossing, although successful, is often insufficient to ensure the penetration of therapeutic molecules into GBM cells, resulting in off-target effects on healthy tissues in the brain that are not intended to receive chemotherapy. For this purpose, various ligands have been investigated to be conjugated on the surface of NPs to improve GBM specificity by recognizing and binding specific or overexpressed features of the GBM cell surface [35,36,37,38]. The most investigated ligands include small molecules such as folic acid (FA), Tf, and Lf, which bind to their respective receptors, and the RGD peptide that targets the αVβ3 integrin. It is noteworthy that, in recent years, most researchers did not focus on the development of novel GBM targeting ligands, but they devoted efforts toward optimizing the combination of already known targeting moieties, NP types, and drugs. This trend probably stems from the repurposing of known ligands and drugs, with well-known toxicity and mechanisms, which can boost the translation of nanosystems from bench to bedside. At the same time, this highlights the intricate co-dependency of NP type, drug used, and ligands to have a positive therapeutic outcome.

There are a few recent papers in which new ligands are proposed for specific GBM targeting, most of them being antibodies or fractions thereof. Guo et al. formulated doxorubicin-loaded PEGylated liposomes (DOPC:DSPE–PEG–COOH) decorated with the ITGA2 antibody for the selective targeting of GBM cells. ITGA2 was proposed as a novel and appealing therapeutic target since it was found to be considerably upregulated in both human GBM and other cancer cells compared to normal brain tissues. The in vitro results confirmed the selectivity of targeted liposomes toward the tumor, being able to cross a leaky BBTB but not a healthy BBB, and exhibiting an IC_50_ value four times smaller than the nontargeted control. The ITGA2 antibody also possessed another potential therapeutic effect by blocking GBM cell migration, further enhancing the antitumoral activity of doxorubicin [39]. Another group engineered biodegradable polymeric poly(lactic-co-glycolic) acid (PLGA) NPs with a cell-surface vimentin antibody M08. M08-NPs showed increased specificity for GBM along with an ability to interfere with tumor cell numbers while promoting healthy cell adhesion when tested in coculture assays. Moreover, a major apoptotic effect and cell death were observed for paclitaxel-loaded M08-NPs compared to nontargeted NPs or free paclitaxel. These results demonstrated the potential of this ligand to be a novel targeting candidate that could be conjugated onto a drug delivery platform for the treatment of GBM [40]. A major limitation to both the articles by Guo et al. and Duskey et al. is that all assays were only performed in vitro, and further in vivo analyses are needed to corroborate these results.

In the framework of tumor-homing peptides, a new targeting ligand (Ser–Ile–Trp–Val/SIWV), derived from an isoform of annexin A3, was discovered by Huh et al. SIWV was coupled with porous silica NPs (SIWV-NPs) in order to selectively deliver an anticancer drug to GBM. Biodistribution analysis in BALB/c mice with xenograft implantation showed successful brain accumulation with a higher presence of silica SIWV NPs at the site of the GBM xenograft. This resulted in a prolonged survival rate and a significant reduction in tumor mass, probably thanks to the caveolin overexpression at the BBB level [41]; however, it is important to note that the GBM tropism was way more evident in vitro compared to the in vivo results, where NPs were also found in other brain tissues [42]. In another study published in 2021, the same team demonstrated that the silica SIWV-NPs could penetrate a human GBM tumoroid more efficiently compared to the same nanoformulation functionalized with the RGD peptide or PEGylation [43]. Similarly to the previous work, specificity resulted particularly enhanced in vitro, but this behavior was reduced in vivo, where silica SIWV-NPs demonstrated preferred accumulation in the GBM xenograft, but they were abundantly found in other brain tissues. While this innovative system holds very high potential for GBM treatment, it is crucial to find novel ligands with significant GBM specificity in order to reduce side-effects on healthy tissues.

Another potential anti-GBM therapy based on peptide-targeted NPs was developed by Hsieh and coworkers. The peptide CTCE9908 was previously used for specific drug delivery in different types of cancers due to its behavior as agonist for the chemokine receptor CXCR4, but never for GBM [44]. In this study, the CTCE9908 was conjugated on lipid calcium phosphate nanocarriers loaded with siRNA, demonstrating effective and efficient delivery of the siRNA both in GBM cell cultures and in vivo in orthotopic GBM mice. This increased uptake resulted in PD-1 ligand gene silencing; however, although there was improved delivery of CTCE9908-NPs in the tumor area compared to nontargeted nanocarriers, a notable biodistribution was found in the kidney and in the liver, which is synonymous to a rapid clearance from circulation and might underline poor GBM specificity [45].

## 4. Dual Targeting of the BBB and GBM Cells

As evidenced by the results presented in the previous section, GBM targeting moieties are crucial to allow for GBM specificity, but their efficacy is often limited in vivo due to the complexity of the biological system, as nanocarriers need to both cross the BBB and be specific toward GBM. Thus, in recent years, two trends have emerged. The first one is the combination of two known ligands, one for BBB crossing and the other for GBM. The second one, instead, involves the functionalization of NPs with a novel single ligand that realizes both BBB crossing and tumor targeting, relying on the expression of common receptors on endothelial and tumoral cells. Here, we discuss some of the work published after 2019.

A sequential targeting approach was proposed by Kuo’s group by developing PEGylated poly(ethylene glycol)–poly(ε-caprolactone) polymeric NPs engineered with wheat germ agglutinin and FA to target the BBB and GBM, respectively (WF-NPs), and loaded with the anticancer drugs etoposide, carmustine, or doxorubicin. The authors demonstrated that the double-targeted WF-NPs showed increased BBB penetration and GBM internalization in vitro, highlighting a synergistic effect of the two ligands that resulted in increased accumulation compared to the single ligands and the nontargeted NPs [46]. While interesting, this study was only limited to in vitro experiments, and an in-depth analysis on the in vivo biodistribution of these NPs is necessary to assess the potential of this strategy to overcome the BBB and specifically target GBM cells.

Wu et al. carried out an interesting study on the surface modification of cabazitaxel-loaded PEGylated nanocrystal liposomes composed of hydrogenated soy phosphatidylcholine and mPEG2000-DSPE. The D-peptide (VAP), agonist of a receptor which is overexpressed both at the BBTB and on GBM cells, and p-hydroxybenzoic acid (p-HA), known to be BBB-permeable, were linked to the surface of liposomes, generating a “Y-shaped” targeting moiety. The double-targeted liposomes resulted in enhanced BBB crossing and GBM spheroid accumulation in vitro compared to both the free drug and the monofunctionalized NPs. When dosed to orthotopic GBM mice, double-targeted NPs were able to significantly cross the BBB, exerting antiangiogenic and apoptotic effects after drug release; however, the authors did not specifically address the GBM specificity, as they did not quantify the amount of nanocarrier or drug in the tumor, but only in the whole brain [47]. This is a crucial point that must be addressed in these types of studies, as it is fundamental to determine the actual specificity of the proposed system.

The popular RGD peptide was used by Hu et al. to formulate tumor-targeted self-assembling nanocarriers. The team synthesized a pH-sensitive doxorubicin prodrug and a derivative of the RGD peptide, which were able to spontaneously form nanocarriers when combined. Specifically, the combination of these two molecules in different ratios led to the selective formation of vesicles or micelles, both showing selective biodistribution to GBM in vivo, with improved anticancer therapy following the release of doxorubicin [48]. More recently, Qi and coworkers combined the targeting ability of the popular RGD peptide and Lf into a drug-loaded DSPE-PEG liposomal nanosystem, finding an optimal RGD:Lf ratio of 3.75:1. The aim was to enhance the performance of the well-established single-ligand-decorated NPs thanks to the ability to bind both the Lf receptor and integrin αVβ3. The expected synergistic effect of RGD and Lf was confirmed by improved transport across an in vitro BBB model and deeper penetration into tumor spheroids compared to the formulations with the single ligands. The brain- and tumor-targeting ability was also demonstrated in vivo, where RGD-Lf liposome administration resulted in an increased chemotherapeutic efficacy of docetaxel, inducing a reduction in the tumor mass, as well as increases in apoptosis and the survival rate of mice [49].

While dual-targeting systems using two known ligands show great promise, the possibility of using a single ligand for dual targeting has also been investigated. With the aim of exploiting the upregulation of LRP1 receptor in both BBB and GBM cells, a novel targeting stapled peptide was synthesized and conjugated onto PEGylated polylactic acid polymeric micelles by Ruan et al. The peptide ST-RAP12, derived from the RAP protein, had a higher LRP1-binding affinity and uptake in endothelial and GBM cells than the non-stapled form. While ST-RAP12 micelles could not reach the brain statistically more than RAP12 micelles, both surface-modified nanocarriers demonstrated a significant increase in brain and GBM accumulation compared to nontargeted micelles. The potential clinical application of this new peptide-modified NPs was supported by an increased survival rate, apoptosis, and angiogenesis inhibition after administration of ST-RAP12 micelles loaded with paclitaxel in glioma-bearing mice [50]. The possibility to use a single ligand for dual targeting represents a great advantage from a technological point of view, as it allows the use of a simpler system, with fewer formulation steps and, consequently, easier characterization and cost-effective production.

Along the same lines, Li et al. synthetized DSPE–mPEG micelles decorated with a peptidic ligand (^D^ATP) to interact with the neuropeptide Y receptor Y1. ^D^ATP was demonstrated to not only promote concomitantly BBB transcytosis and glioma targeting of micelles, but also to improve these processes compared to the functionalization with ATP in the L configuration (^L^ATP). The in vitro data highlighted an increased BBB crossing compared to Angiopep-2- and RVG29-modified micelles, two well-known targeting moieties. Furthermore, the favorable properties of ^D^ATP-micelles improved the application of photothermal therapy in GBM mice, resulting in a greater therapeutic effect [51]. Interestingly, the authors claimed that ligand density led to different amounts of uptake in GBM cells; however, the ligand density was not quantified, and they only referred to the amount of ligand used for different formulations. This is a crucial point, as discussed in detail in Section 6, that demonstrates the importance of a thorough characterization of the surface of targeted NPs, as well as the lack of techniques available to succeed in this aim.

Starting from a viral glycoprotein, a derivative peptide of 15 amino acids called RVG15 was recently demonstrated to be a novel and efficient targeting peptide for BBB crossing, since it is able to bind the nicotinic acetylcholine receptor [52]. This peptide was conjugated onto the surface of soybean phosphatidylcholine, cholesterol, DSPE–PEG liposomes loaded with cholesterol-conjugated paclitaxel, providing BBB permeability and selectivity toward GBM cells [53]. Indeed, RVG15 liposomes improved paclitaxel delivery and efficacy compared to nontargeted nanosystems; not only could RVG15 liposomes accumulate in the brain 1 h after administration in vivo, but they were also abundantly present in tumors. The interference with GBM progression was ascertained through the inhibition of tumor growth and metastases formation. Unfortunately, the authors did not perform statistical measurements to assess the prevalence of liposomes in the tumor tissues compared to surrounding healthy tissues in the brain, which is a critical evaluation to determine the GBM specificity of this interesting dual ligand.

Another ligand synthesized for two-in-one targeting purposes was the tetrapeptide mnRwr (mn), which should interact with the integrin αVβ3. In order to evaluate its performance, the authors compared mn with the well-studied c(RGDyK) peptide. BBTB transcytosis and tumor spheroid penetration were comparable for the two ligands. In contrast, mn-liposomes exhibited improved biodistribution and pharmacokinetics in vivo, resulting in a greater accumulation in the tumor site of xenograft-injected mice, as well as increased therapeutic efficacy against GBM [54].

Another innovative strategy was proposed by Huo et al. Exploiting the high Tf concentration in the blood and the upregulation of Tf receptors in both brain endothelial and GBM cells, covalent organic framework nanospheres, composed of PEI-coated mesoporous silica nanospheres, were decorated with the peptide T10, which has a high affinity to Tf. After systemic administration, a Tf-based corona on the surface of NPs was generated in the bloodstream due to the strong Tf-T10 association, allowing for the specific endogenous interaction of Tf-coated NPs with Tf receptors. The BBB- and GBM-targeting ability was demonstrated after in vivo administration, corroborated by a decrease in IC_50_ value and a prolonged mice survival rate compared to the free drug, the nonmodified formulation, and the commercial liposomal doxorubicin Caelyx^®^ (Baxter Holding B.V. Utrecht, The Netherlands). Interestingly, T10-modified NPs presented a prolonged release profile for doxorubicin and, most importantly, did not involve the action of the efflux pumps [55]. This study represents an interesting and innovative approach toward tumor targeting, as it exploits endogenous mechanisms by directing the formation of the protein corona to allow for a more specific and nonimmunogenic brain and GBM targeting.

These studies displayed groundbreaking results in the field by simultaneously overcoming what are considered two of the major barriers to GBM delivery and treatment. Table 1 summarizes the selected nanosystems. While showing improved results, the characterization and in vivo analyses are still critical missing steps in their path toward a true GBM treatment, which becomes even more complicated in animal models with the biological differences in the tumor vicinity, as discussed in the next section.

## 5. Targeting the Tumor Microenvironment (TME)

So far in this review, novel strategies to target the BBB or GBM cells have been discussed; however, there is another option that has recently entered into play to try to combat cancer. The TME has taken the spotlight in recent years as a promising target as it plays a pivotal role in tumor growth and in the formation of metastasis. Indeed, it concentrates solutes and cells that can trigger the epithelial-to-mesenchymal transition of epithelial cells, which can ultimately lead to the formation of metastasis [62]. All cancers host a TME which could vary between patients and cancer types/progression, but the study of the differences and intricacies between each different TME is still in its infancy and not known well enough to make distinctions. Therefore, targeting the TME cannot be considered GBM-specific, but will surely play a critical role in the improved treatment of GBM, as well as other cancer types. Moreover, in recent years, the possibility of studying TME characteristics has been supported by the big improvements in microfluidic-based techniques; with this technique, it is possible to regulate the interstitial flow, rheological properties, and nutrient and oxygen concentration, finely mimicking the physiological characteristics of the TME [63]. This allows us to have more accurate models with faster and more accurate analyses that will translate into more effective systems. In order to exploit the characteristics of the TME to reduce tumor growth, NPs have been shown to be particularly efficient, thanks to their specific targeting potential [64]. Over the last decade, the number of studies on the TME has exploded; in 2021, the number of review articles on this topic seemed to outnumber the research articles, allowing readers to look up and understand the idea of the TME and how it can be taken advantage of for enhanced chemotherapy. Therefore, in this section, we only give an update on the most recent studies (2022) regarding TME targeting and how they are advancing potential therapeutic strategies.

One of the oldest, most investigated, and well-known ways to target tumors linked to the TME characteristics is the enhanced permeation and retention effect (EPR). The EPR effect was first observed in the 1990s, and it has led the production of nanomedicines with a size around 200–300 nm to facilitate extravasation and accumulation at tumor sites [65]; however, recent considerations that the EPR effect is minimized in humans compared to rodents, as well as its heterogeneity from patient to patient or disease to disease, have made it more evident that the EPR effect alone cannot ensure the specific accumulation of NPs in tumors or successful translation to a clinical setting [66,67,68,69,70]. For this reason, NPs have been further engineered to enhance their efficacy [71]. Targeting moieties toward TME components can be used to enhance the efficacy of NPs that are already present and trapped near the cancer cells due to the EPR effect. For example, to further facilitate the penetration of NPs in the matrix of solid tumors, their surface can be decorated with ligands that target cells of the extracellular matrix (ECM), such as pericytes, endothelial cells, or cancer associated fibroblasts [72,73,74]. To target the ECM, several studies have investigated the use of collagenase or hyaluronic acid to coat chemotherapeutic-loaded NPs. While the use of hyaluronic acid can promote extravasation following interaction with the CD44 receptor [75], the use of collagenase was recently demonstrated by Shukla et al. to improve ECM penetration and accumulation at the tumor site of biocompatible albumin NPs loaded with gemcitabine. The efficacy of these coated NPs was tested in tumor spheroids, demonstrating more efficient delivery of gemcitabine to cancer cells compared to NPs without collagenase [56].

The high concentration of growth factors that occurs at tumor sites has also been investigated for NP targeting. In particular, the epidermal growth factor (EGF) is one of the most researched moieties with which to decorate NPs for cancer therapy. While most of the previous strategies to target the EGF receptor were based on the use of monoclonal antibodies, such as the anti-EGF receptor monoclonal antibody cetuximab, on the market as Erbitux^®^ (Merck Healthcare KGaA, Darmstadt, Germany), the endogenous EGF molecule (6 kDa) was recently proposed as a smaller and easier molecule to use as a targeting ligand to be conjugated on the surface of NPs [76,77]. Skóra et al. confirmed the possibility of selectively delivering silver NPs to cancer cells by loading them into EGF-decorated liposomes that were able to induce toxicity in cancer cells in vitro [57]. Growth factors and cytokines are also the target of the research published by Zhang et al. in June 2022; here, the authors validated the use of gold NPs to inhibit the tumorigenic phenotype and tumor growth both in vitro and in vivo, highlighting the crucial role of soluble hormones in the TME in tumor progression [78]. Another hormone that has been used to target cancer cells is the vascular endothelial growth factor (VEGF). VEGF receptors are highly overexpressed in most solid tumors to allow for a fast growth of cancer cells. Thus, using VEGF as a targeting moiety for the TME has represented one of the most promising targeting strategies for a long time [79]. Unfortunately, it has been evidenced that tumors are often insensitive to antiangiogenic treatments with anti-VEGF therapies. This phenomenon has been mostly observed in highly innervated tumors, revealing the crucial role of tumor-associated nerves (TANs) in the efficacy of anticancer treatments [80,81]. Treating tumors with VEGF-targeted NPs could induce a response from TANs with production of neurosignals that might restore the angiogenic behavior of cancer cells, canceling the efficacy of the treatment [82]. This crosstalk highlights the complexity of the TME and the need for more in-depth studies of the conditions that can occur in tumor sites, together with the need of novel systems that can address more than one target with a single administration.

Once in the TME through the EPR effect or active targeting, NPs can also be designed to take advantage of some of the characteristics of the TME by passive targeting. The TME is characterized by acidic, hypoxic, and oxidative conditions, which have been widely addressed as targets for stimulus-responsive NPs [83,84,85,86,87]. In January 2022, Wei et al. proposed a system for diagnostic purposes where a pH-sensitive moiety was conjugated on the surface of Fe_3_O_4_-based probes. This moiety was demonstrated to be able to interact with cancer cell membrane at acidic pH, highlighting the presence of cancer cells by PET/MR imaging [88]. Koo et al. optimized a system based on copper–iron peroxide NPs which can degrade in acidic conditions. This system, designed for chemodynamic therapy of solid tumors, produced oxygen, relieving the hypoxic state, and released Fe ions, which improved MR imaging as a contrast enhancement [89]. It is also important to underline that inorganic NPs, while demonstrating impressive results in the treatment and possibility for more precise or early-stage diagnostics, are also known to have issues with toxicity or the inability to be cleared from the body, which might represent a limiting step in the transition toward the clinic and that must be taken into consideration when designing novel NP-based strategies [90,91]. Overall, the pH or hypoxic reactive lipidic or polymeric NPs were unfortunately less published this year compared to previous years, probably due to the lower specificity of this type of targeting than receptor-mediated targeting.

As previously mentioned, activating the immune system to recognize tumors became a novel line of research against tumor cells in the last decade; however, this approach often resulted ineffective, as the TME can block immune recognition [92]. Over the last year, several studies still tried to overcome this barrier by using NPs to modulate the immune system and activate it against tumoral cells [93]. For example, NPs have been used to deliver siRNA or mRNA to cancer cells to modulate the expression of specific proteins, such as Her2. Downregulation of Her2 recently resulted highly effective in promoting cell death in breast cancer models, exhibiting a synergistic effect with taxane [94]; however, Her2 reduction leads to the production of cytokines that might promote tumor recurrence [95]. Moreover, cancer cells can overexpress antiphagocytic molecules such as CD47, ultimately inhibiting the inflammatory response and facilitating immune escape of cancer cells [96]. Therefore, one of the most recent and innovative approaches is to act on the TME in order to block the inhibition of the immune response, to finally allow the activation of immune cells towards tumors. This strategy, called immune checkpoint blockade (ICB) therapy, is now on the rise, with several reviews highlighting its potential; however, most of them focused on the lack of research studies, due to the inherent difficulty and complexity of this kind of approach [72,73,97,98]. Modulation of the TME for immunotherapy and ICB therapy can be performed by addressing soluble cytokines, enzymes, and other proteins e.g., CTLA-4 protein and PD-1 receptor, as well as cells such as T-cells, cancer-associated fibroblasts (CAFs), and tumor-associated macrophages (TAMs).

Macrophages in the TME usually have an M2 phenotype, meaning that they have an inherent anti-inflammatory activity. Therapies targeted to TAMs can include the repolarization of anti-inflammatory M2 macrophages to the proinflammatory M1 phenotype and inhibition or elimination of activated TAMs. Feng et al. recently proposed a drug delivery system by engineering an albumin-based NP with scavenger receptor A and SPARC protein which targeted both TAMs and tumoral cells, respectively. The group demonstrated that these targeted NPs were able to eliminate TAMs from the TME, leading to an increase in inflammatory cytokines that would ultimately promote immune recognition of tumoral cells [58]. Wang et al. recently developed ultrasmall Cu_2−x_Se inorganic NPs coated with a biomimetic cell membrane for ICB therapy of GBM. These NPs were able to polarize the macrophages to an M1 phenotype and decrease the expression of the PD-1 ligand. Moreover, after treatment with their NPs, they noticed an increase in memory T cells in the spleen, which could possibly prevent GBM recurrence through the protection by the immune response [59]. Similar results were obtained by Alghamri et al. with synthetic protein NPs modified with a transcytotic peptide and loaded with an inhibitor of the CXCL12/CXCR4 signaling pathway, which is associated with tumor growth and progression. This NP drug delivery system, when coupled with radiation therapy, was able to block the signaling cascade, resulting in induced immunological cell death and the proliferation of memory cells, lowering the risk of recurrences in a GBM mouse model [99].

Another interesting approach is to target regulatory B cells (Bregs), which have been recently proposed to be responsible for the attenuation of the antitumoral immune response, as they can upregulate the production of interleukin 10 and the PD-1 ligand [100]. This strategy was investigated by Shen et al. using lipid-protamine NPs to load plasmid DNA encoding a CXCL13 trap to reduce the differentiation of Bregs. The DOBP- and cholesterol-based lipid–protamine–DNA (LPD) NPs developed by the team successfully reduced tumor growth, showing the pivotal role of Bregs in tumor progression [101].

To enhance the efficacy of any treatment targeted to the TME, some interesting approaches were published this year using dual systems that can exploit two of the cited targeting strategies with a single nanosystem. To this end, Ding et al. proposed the formulation of an alginate hydrogel loaded with two separate nanosystems: (1) porphyrin-modified Fe_3_O_4_ NPs, sensitive to near infrared light for photodynamic therapy and able to produce reactive oxygen species (ROS); (2) albumin-based NPs, conjugated to an antibody against the PD-1 ligand via a ROS-sensitive linker that could be cleaved in the TME containing high levels of ROS. This complex system was demonstrated to have high therapeutic efficacy by combining the positive effects of photodynamic therapy, ROS induction, and immunomodulation [60]. Another dual strategy system was presented by Sun et al., where platinum NPs stabilized with phenylboronic acid were co-assembled with dextran-coated NPs loaded with sotuletinib. The linkage between the two was a pH-sensitive borate ester, which allowed the system to disassemble at the acidic pH of the TME. After disassembly, platinum NPs were able to penetrate in the most internal layers of the tumors and induce cell death due to the release of Pt^2+^, while sotuletinib was able to induce the depletion of TAMs, reversing the immunosuppressive microenvironment [61].

The TME has become a hot topic in the research field, and targeting its variable components has become a huge area of research which holds great promise for understanding and better controlling the way in which a tumor survives, grows, and re-emerges after treatment. While it is present in all tumors, a better understanding the TME will play an even more critical role in treating the more difficult-to-reach tumors, such as GBM. With the help of nanotechnologies, utilizing these differences around the tumor masses could help improve early-stage diagnostics and late-stage treatments; however, we are still very naïve about the interplay and complexity of the TME and how to take advantage of it to its full potential. Reviews continue to describe the ideas and key points of the TME, but a huge amount of research is needed in order to optimize a system that combines its numerous components for a complete and therapeutically relevant system.

## 6. Surface Characterization

As discussed in previous sections, surface functionalizations of NPs are key to achieve targeted delivery of chemotherapeutics, especially for GBM and brain tumors. Unfortunately, the characterization of the surface of NPs is often overlooked, due to the lack or high cost of the techniques used, as well as the difficulty of specifically looking at only the surface and not the whole nanosystem; however, surface characterization is a crucial step of the development of a nano-drug delivery system. From a biological point of view, the surface is responsible for the interaction with the target, and its characteristics can influence the composition of the protein corona, the clearance time, and the toxicity of the system. From a technological perspective, it is important to understand the surface composition of NPs in order to maximize the efficiency of the engineering process, while also ensuring the formulation of reproducible NPs that can be translated into an industrial and clinical setting.

Paramount parameters to describe NPs are size, surface charge, and shape, along with porosity, surface chemistry, crystalline structure, purity, drug loading, hydrophobicity, etc. Currently, several technologies are available for the characterization of NPs [102,103], with the choice of the method depending on the sample characteristics and the final application [104]. More often than not, it is difficult to achieve a thorough characterization of a NP system due to several challenges, the most neglected aspect being the surface characterization [105]. The lack of accurate characterization of NP surface could be one of the reasons for undesired biodistribution in off-target organs and/or rapid clearance. Indeed, the fate of the NPs in the complex biological environment after administration both in vitro and in vivo is determined by the interaction of the surface with the various biological components. It is well known that surface chemistry has implications in solubility, stability, biocompatibility, and pharmacokinetics of NPs, along with influencing the internalization and the ultimate efficacy of the nanosystem [106]. One of the most important aspects to be determined when characterizing an NP system is the ligand density. Depending on ligand density, two trends of cellular uptake can be identified for a single ligand: (1) optimum density with a plateau, and (2) optimum density with a maximum [107]. The identification of a behavior over the other would lead to a more conscious interpretation of biological results. Moreover, this would be pivotal information that could greatly improve the design and optimization of NPs with the optimal density of ligand to exert its targeting activity, as well as reduce the waste of materials during the formulation process.

**Figure 2:**
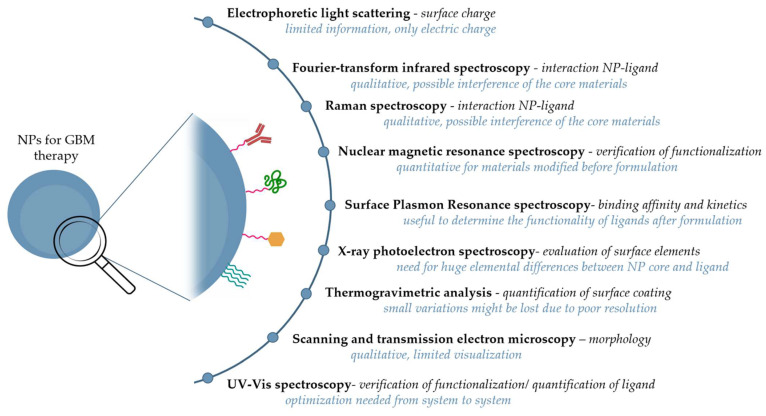
Techniques used for surface characterization of NPs for GBM therapy. Abbreviations: GBM—glioblastoma multiforme; NPs—nanoparticles; UV-Vis—ultraviolet-visible.

Unfortunately, many papers on NPs for GBM treatment lack an adequate surface analysis before moving on to animal studies. If present, this could help prevent therapeutic failure, or it would allow higher NP accumulation at the tumor site. The surface characterization can be carried out using both classical and advanced tools that, however, do not allow a single-level analysis, but only in bulk [108]. Because of the complexity of NPs and the information obtained from each technology, the preferable method to analyze the surface of NPs would be a multidisciplinary approach. Different techniques should be used to extract complementary information with the purpose of having a snapshot of the system that is as detailed as possible before proceeding with further in vitro and in vivo tests. An overview of analytical tools used to obtain information about the surface features of NPs formulated for GBM therapy is shown in Figure 2.

### 6.1. Electrophoretic Light Scattering

One of the two most commonly studied NP parameters throughout the literature is the surface charge (the other being hydrodynamic diameter). By exploiting the electrophoretic light scattering, the zeta potential can be measured in an easy and fast way. This value not only provides information on the surface charge of NPs in suspension, but can also help predict the stability of the formulation, as colloids with a zeta potential close to neutrality often tend to form aggregates during storage [109,110]. Moreover, the zeta potential is a crucial parameter when considering the toxicity of nanosystems, as it is well known that cationic NPs tend to be quickly cleared from the bloodstream and induce toxicity after administration [111]. In several studies, surface charge measurements at different steps of the formulation are used as a control for the effective functionalization of NPs; however, this parameter often does not give an indication of the amount of ligand. In fact, a shift in the zeta potential is generally considered significant only for huge changes, and its use as an indicator for successful modification is often limited to the case of coated NPs [112,113]. On the contrary, ligands such as peptides, small molecules, or antibodies hardly ever induce a significant shift in the zeta potential, due to the small amount added to the formulations. Moreover, shifts in the zeta potential are only qualitative, and they do not provide information about the amount of ligand or coating molecules present, nor whether they are chemically conjugated or nonspecifically adsorbed on the surface, or whether they are present in the correct 3D orientation, which is a critical aspect of antibody-based targeting strategies. Unfortunately, quantification of the targeting ligands on the surface of NPs is often a long and difficult task that requires multiple techniques.

### 6.2. Fourier-Transform Infrared Spectroscopy

One of the most widely used techniques for assessing the functionalization of anti-GBM NPs is Fourier-transform infrared spectroscopy (FT-IR). FT-IR is a vibrational, nondestructive method that provides structural and chemical information of a sample by using radiation in the infrared field. The positions and the intensity of the bands in the IR spectrum allow a qualitative analysis, indicating the presence of the ligand interacting with the NP components. Over the years, FT-IR has proven to be a versatile and powerful tool for material characterization [114,115,116,117]. In [118,119,120,121,122,123], the effective coupling of the targeting moiety was evaluated through the direct spectral comparison among the whole NP formulation, the nontargeted one, and/or the ligand. The attribution of specific peaks made it possible to identify the contribution of the ligand in the IR spectrum of NPs. Unfortunately, other components in the NP or the drug itself might interfere with the signals coming from the ligand; thus, this technique does not give quantitative information about the NP surface, but only qualitative. Another major limitation of this technique for surface characterization lies in the fact that the IR light might penetrate into the matrix of the NP [124]. This is a crucial aspect especially for pre-conjugated ligands that might end up being entrapped in the matrix but not displayed on the surface, e.g., in the case of pre-modified lipids that during the formation of a liposome could be faced either in the internal phase or onto the surface. In this case, FT-IR would give positive results, but it would not take into account the real 3D disposition of the ligand.

### 6.3. Raman Spectroscopy

Complementary to IR, Raman spectroscopy (RS) relies upon the inelastic scattering of light and provides a molecular fingerprint of the analyzed target. Thanks to the possibility of obtaining qualitative and quantitative information with a low amount of sample in a few minutes through label-free analysis, RS and surface-enhanced Raman spectroscopy have found application in the pharmaceutical and biological field [125,126,127]. Although RS offers many advantages, its application for NP characterization is not widely exploited. For example, Sahli’s group verified the formation of a complex among PEGylated gold NPs, temozolomide, and gemcitabine via RS, where the decrease in the peak intensity at 1292 cm^−1^ in the presence of the drugs denoted the reaction between COO^−^ and NH_3_^+^ groups indicating successful conjugation [128]. Similarly, RS was also used to assess the bond of cisplatin to gold nanopeanuts developed as a dual therapeutic platform against GBM [129]. Difficulties in the use of this technique lie in the complexity of Raman spectra interpretation since the technique gives an overview of the presence, concentration, and interaction of all the molecules present in the sample; however, the comparison between formulations that differ in only one component can help identify the contribution of the molecule of interest in the Raman spectrum. Lastly, similar to FT-IR, when NPs are functionalized prior to formulation, the possible penetration of the laser in depth of the NPs can generate signals not specifically related to the ligand present on the surface, but also inside the matrix.

### 6.4. Nuclear Magnetic Resonance Spectroscopy

Nuclear magnetic resonance spectroscopy, also called NMR, is a robust and informative platform to obtain structural information about molecules. Taking advantage of nuclear spin variation, NMR is able to identify the type and the abundance of isotopes in both liquid and solid samples. Therefore NMR can provide insight into surface features and modifications [130,131,132]. For example, NMR spectra were used to confirm the successful conjugation of Angiopep-2 on polymers for NP formulation either by the lack of the N-hydroxysuccinimide peak or by the presence of Angiopep-2 peaks on the polymer spectrum [133,134]. Another approach that can be followed is the attribution of the chemical shift. Similarly, Minaeia et al. ascertained the binding of FA to polymeric NPs through the identification of protons related to the grafted molecule, while Vilella et al. confirmed the conjugation of a BBB-crossing peptide to PLGA to be used to formulate brain-targeted NPs [135,136]. NMR spectroscopy can be used to quantify the conjugation yield of a ligand, indicating to which extent the material to be formulated into NPs has been linked to the targeting moiety. However, similarly to the techniques previously discussed, this information is not indicative of the actual presence of the targeting ligand on the surface of the system.

### 6.5. Surface Plasmon Resonance Spectroscopy

In addition to the quantification of the ligand present on the surface of a nanosystem, it is crucial to verify whether it still retains the ability to bind its receptor after coupling with NPs; to this aim, the nanoformulation can be analyzed by surface plasmon resonance spectroscopy (SPR). SPR is a highly sensitive optical technique used to study the binding affinity and kinetics between an analyte in solution and ligands spotted on a gold chip in real time, without the need for any labels. Since the evanescent wave propagates for about 200 nm from the chip surface, SPR is a suitable tool for the analysis of nanosystems [137,138,139]. Using SPR, Gries et al. compared the binding kinetics between the free peptide KDKPPR and the peptide-based NPs against GBM, not only demonstrating that the coupling did not impair the activity of the ligand, but also finding a higher K_D_ after the coupling of the targeting moiety with NPs [140]. The same rationale was used in another study, in which the Tf binding efficiency after coupling to chitosan NPs was also quantified through inductively coupled plasma optical emission spectroscopy [141]; however, the authors did not exploit the multiplexing characteristic of SPR. Indeed, no receptor apart from the Tf receptor was immobilized on the chip in order to evaluate the specificity of the binding, resulting in incomplete information, which might be useful to predict the efficacy of this nanosystem. Notwithstanding the useful information that can be obtained from sensorgrams about the optimization of NP and their potential in vitro and in vivo efficacy, SPR is not widely present in laboratories. Limitations may be due to the cost of gold chips on which ligands are spotted, along with the optimization of the surface chemistry of the chip for ligand immobilization, necessary to obtain reproducible substrates with desired characteristics.

### 6.6. X-ray Photoelectron Spectroscopy

Among the available techniques, X-ray photoelectron spectroscopy (XPS) represents a valuable tool for surface characterization. According to the photoelectron emission process, XPS reveals the elemental composition of the outermost layer (within 10 nm) of the material after the irradiation of X-rays; however, a possible limitation is the ultrahigh-vacuum condition necessary for the analysis, which might be incompatible with the material used for the formulations. Over the years, this sensitive electron spectroscopy has been used for the surface characterization of almost every material [142]. Since all elements except H and He can be detected, it is possible to follow the variation in NP surface composition before and after the functionalization procedure. For example, the variation of C concentration and its signal reflected the formation of the metal–organic framework layer on Fe_3_O_4_ NPs [143]. With the same aim, Venditti et al. analyzed Au-NPs coated with two different thiol-bearing moieties that may serve as linkers for future ligands. The XPS analysis was able to distinguish the signal of the gold atoms belonging to the NP core, from that relative to the surface atoms. This allowed measuring that around 20% of the gold atoms on the NP surface were covalently bonded with sulfur of the two moieties [144]. One of the major limitations to the use of this technique for the characterization of surface-modified NPs is that this is particularly efficient in the determination of metal ions; in general, the more the material of the NP and the ligand are chemically different, the more this technique is useful and efficient. Unfortunately, organic NPs are often functionalized with organic moieties, e.g., liposomes, PLGA, or chitosan NPs, conjugated with peptides, antibodies, or small molecules. In these cases, the use of XPS is greatly limited by the high elemental similarity of the components, which hampers the possibility of using this method for surface characterization.

### 6.7. Thermogravimetric Analysis

Thermogravimetric analysis (TGA) is a technique that correlates the mass of a sample with the increasing temperature, and it is a commonly used technique for a quantitative measurement of a sample with application in the study of nanomaterial structure [145,146,147], as changes in the specimen mass indicate the loss or transformation of one or more components, particularly related to the adsorption or desorption of coating layers; however, no indication of the nature of the material lost is given. Exploiting TGA, Świętek et al. studied the formation of multiple coatings on the surface of silica–Fe_2_O_3_ NPs, using tannic acid and chitosan. While this technique allowed authors to measure the amount of water present in the sample, which could be related to the number of hydrophilic components in the coating layers, it was necessary to couple this analysis with other techniques to have an overview of the sample. The formation of the layers was evaluated in parallel with FT-IR and zeta potential, and the presence and intensity of specific IR peaks and the variation in surface charge were considered as markers of the successful coating procedure [148]. The analysis of the coating formation was also verified by Shahein et al. after the formulation of mesoporous silica NPs covered with two different shells: one made of chitosan–stearic acid (CS) and the other made of whey protein–gum Arabic (WA). In this study, the amount of CS and WA forming the coating was deducted from the mass variation between the nanoformulation with and without the coating layer [149]. While interesting, this approach requires the instrument to have a very high resolution, so as to measure small variations in the case of NPs where the amount of material used for the coating is small.

### 6.8. Scanning Electron Microscopy and Transmission Electron Microscopy

**Figure 3:**
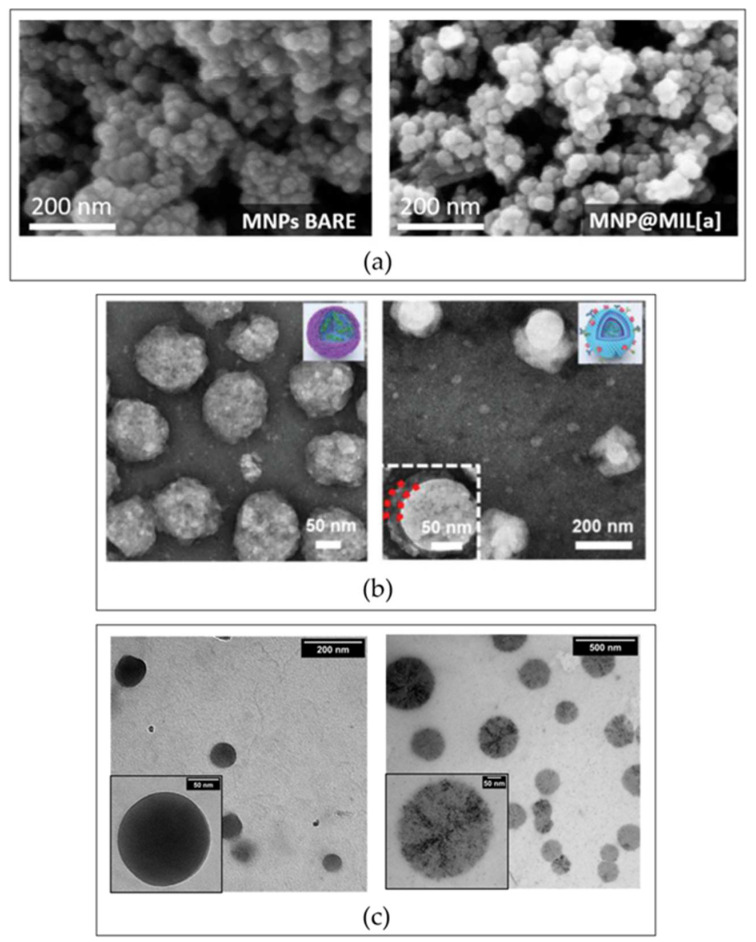
Morphological characterization of surface-modified NPs. (**a**) Fe_3_O_4_ magnetic NPs covered with a metal–organic framework. SEM images of bare (**left**) and decorated (**right**) NPs. Adapted with permission from Pulvirenti et al. [143] (IJMS, MDPI, 2022). (**b**) TEM analysis of biomimetic NPs made up of siRNA complexed polyethyleneimine xanthate before (**left**) and after the cell membrane coating (**right**). Adapted with permission from Zhang et al. [154] (Adv. Funct. Mater., Wiley, 2022). (**c**) TEM images of hyaluronic acid NPs pre (**left**) and post (**right**) modification with Angiopep-2. Adapted with permission from Costagliola di Polidoro et al. [155] (Cancers, MDPI, 2021). Abbreviations: NPs—nanoparticles; SEM—scanning electron microscopy; TEM—transmission electron microscopy.

Scanning electron microscopy (SEM) and Transmission electron microscopy (TEM) are well-established tools for imaging in the field of material characterization. SEM creates a 3D image by detecting the reflected electrons, whereas TEM uses the transmitted electrons to provide a 2D image. As a result, SEM can help yield more detailed information about the sample surface while TEM can provide information about the internal structure. For both techniques, the high resolution enables visualizing specimens at the nanometer level; hence, they have found application in NP characterization by allowing the study of shape and size [150,151,152]. Numerous studies have used SEM and TEM images to better characterize and understand the influence of surface functionalization on morphology, porosity, and surface area of nanocarriers. Ghaferi et al. carried out SEM analysis at different stages of the synthesis and formulation of drug-loaded PEGylated liposomes. Images, however, did not provide evidence of surface changes, but only of the retention of the spherical shape and the monodispersity after the decoration with PEG molecules [153]. Similar information was obtained by Pulvirenti and coworkers analyzing Fe_3_O_4_ NPs grafted with a metal–organic framework layer (Figure 3a) [143]. Indeed, even if SEM is considered a valuable technique for evaluating the features of material surfaces, no differences in texture following the modification were observed in the studies mentioned above. On the other hand, TEM images of biomimetic NPs developed by Zhang and coworkers highlighted a core–shell architecture after coating an siRNA-based complex with melanoma cell membrane (Figure 3b) [154]. In another example, differences in the TEM images of hyaluronic acid NPs revealed a structural modification after conjugation of Angiopep-2 on their surface (Figure 3c) [155]. While both of these techniques are fundamental and critical for demonstrating a full and intricate characterization of reproducible and stable NPs, authors often miss the description of the differences pre and post modification. This major flaw can be noted not only when commenting on coated NPs, but even more when discussing the characterization of NPs modified with small ligands, where the characterization is even more difficult. A general idea of the effects during the modification process can be determined, but the specific features in terms of location, quantity, or distribution of the ligands are still outside of the resolution range of the general techniques, and they must be improved to have a true knowledge of how these variables could affect the biological and pharmaceutical effects of NPs.

### 6.9. Other Methods

A broadly used technique to characterize NPs is UV/Vis spectroscopy. When applied to surface-modified NPs, this method can help understand if the functionalization has been successfully achieved, by evaluating the absorbance change or band shifting occurring after surface modification. These results are particularly evident in the case of inorganic NPs. In this view, Wang et al. developed maleimide PEG-modified CuS NPs with the aim of combining hyperthermia with immunotherapy for cancer therapy. The absorption spectra of PEG-coated CuS NPs were analyzed using a UV/Vis/NIR spectrophotometer, and the spectra of PEG-functionalized NPs showed a different intensity of absorbance compared to the nonfunctionalized NPs, indicating that the surface functionalization was successful [156]. Similarly, Lesiak and coworkers performed a surface study of iron oxide NPs functionalized with different biocompatible molecules adsorbed on the surface, such as succinic acid, oxalic acid, L-arginine, citric acid, and glutamic acid. They demonstrated that the surface adsorption of these organic molecules had an impact on the UV/Vis spectra of iron oxide NPs, producing a change in both the intensity and the wavelength of absorbance of the modified NPs compared to the nonmodified ones [157]. Those two studies, as well as others in the recent literature, indicate that the UV/Vis characterization of surface-modified NPs is a valid tool in the characterization of inorganic NPs [158,159,160]; however, many researchers demonstrated that organic surface-modified NPs can also be characterized using this technique. This is possible by exploiting the ability of some organic molecules to react with UV/Vis-detectable substances. For example, the amounts of protein- and peptide-based ligands can sometimes be assessed by specific colorimetric assays. Due to the low sample volume required and the simplicity, the BCA assay is the most widely used. This is an indirect ligand quantification which involves measuring the free ligand at the wavelength of 562 nm, meaning that the separation of formed NPs from nonconjugated ligand is required [46,161,162,163]. The purification is often a limiting step during formulation of NPs, and it requires thorough optimization to ensure that the method chosen does not cause NP destabilization and ligand detachment, which would interfere with ligand quantification. Unfortunately, in addition to the purification, the BCA assay is only applicable to a limited class of ligands, and must be properly optimized for each molecule.

An interesting strategy is the combination of size-exclusion chromatography and ultrahigh-performance liquid-chromatography as a novel single-step methodology for ligand separation and quantification. With this strategy, Gazaille et al. could separate lipid nanocapsules decorated with a tubulin-binding peptide from the unconjugated ligand, thanks to this size-exclusion chromatography step. Liquid chromatography was consequently used to quantify the amount of ligand on the surface [164]. Although this approach results very attractive by combining a purification and quantification method, it is important to note that this type of separation is often only possible for water-soluble ligands, as size exclusion gels are often incompatible with elution in solvents, limiting the number of ligands that can be analyzed. Moreover, analysis via liquid chromatography often relies on UV or fluorescence detectors, and some ligands might need an additional derivatization step to be visualized, thus complicating the quantification protocol.

## 7. Conclusions

To date, the multidisciplinary therapeutic approach for GBM has not led to the expected effects needed for a functional treatment, with only slight improvements in the prognosis and life expectancy of the patients, which remains very poor. Thanks to several advantages, NPs have taken the spotlight as novel and more elegant methods to create therapeutics to fulfill the unmet clinical needs against hard-to-treat diseases. With these successes, scientists have exploited these advantages to try to improve GBM treatments as well; however, the fast growth rate, invasiveness, shift in biological microenvironment, resistance to drugs, and vast variability of GBM from patient to patient have greatly hampered their effectiveness to deliver chemotherapeutics to the tumor site and create a system with potential clinical applications. For this reason, more advanced targeted NP systems and early detection options are being designed and researched each year.

Currently, the most studied method to improve GBM treatments using NP delivery systems is conjugating ligands onto the surface of the NPs in order to exploit ligand–receptor interactions to improve specific drug delivery to the GBM tumor and limit off-target effects. Over the years several types of ligands that act on different targets have been involved for this purpose; however, oftentimes, as in the case of Tf and its receptor, they are ubiquitous, and the true selectivity is not up to par. Novel and improved ligands are being sought out to be more specific, even at the early stages of disease progression.

In the case of GBM, the BBB is still considered one of the major barriers to successful chemotherapeutic delivery. Therefore, many new GBM-targeting studies do not actually look at true GBM targeting, but attempt to improve the rate of BBB crossing in order to improve GBM treatment. While this is critical for a therapeutically relevant NP against GBM, it still lacks specificity within the central nervous system and can be subject to toxicity to the local healthy cells. Therefore, other ligands that specifically target the GBM cells over healthy cells are required. These ligands would improve selectivity and safety profiles, but these studies are often limited to in vitro studies, or the potential GBM ligands lack the ability to cross the BBB. To this end, the major portion of recent research articles have explored co-targeting with both BBB and GBM ligands incorporated into a single NP system. While this will drastically improve both delivery amounts and specific toxicity against GBM while maintaining better safety profiles for healthy cells, the complexity of simultaneously formulating, characterizing, and testing the interaction of two ligands is a huge logistical barrier that needs improved methodologies and assays to properly understand and utilize the results. On the other hand, some researchers have proposed novel ligands that might be able to simultaneously promote BBB crossing and display GBM specificity; these ligands would greatly improve the therapeutic efficacy of targeted NPs by ensuring targeting with a single moiety. However, this type of investigation is still in its infancy due to the difficulty of finding GBM specific ligands that allow BBB crossing, as well as optimizing and analyzing both effects contemporaneously.

Actively targeting the BBB and tumor cells with ligands has always been the staple of novel research, but another option has recently presented itself. Researchers have discovered that many targeted systems do not work due to the ability of the tumor cells to modify their TME in order to block the immune response or degrade NP systems due to factors such as acidic pH, high ROS levels, or the presence of solutes and cells that hamper immune recognition. Therefore, instead of directly targeting the cells, the possibility to utilize these variations in the tumor environment to activate the NPs, control drug release, block tumor metastasis, and minimize the immune system inhibition is being researched. Huge numbers of reviews are being published on this topic each year, but our true experimental understanding of the TME for each type of cancer and its variability from patient to patient is still lacking. This will require vast research and comparisons between systems in order to elucidate the true nature and importance of these factors in combating GBM.

NPs targeting the BBB, tumor cells, or TME are being designed and researched in abundance for their ability to combat GBM in highly advanced in vitro or in vivo models; however, a critical component of the research is oftentimes overlooked. While aiming to combat or cure GBM, many times, the characterization of the NP vehicle is overlooked, performing the bare minimum of size, polydispersity, and surface charge characterizations. In order to improve these targeted systems, it is critical to standardize a higher level of surface characterization using various intricate techniques to determine the amount, position, distribution, and availability of these targeting ligands on the surface of the NPs, as well as to correlate those results with how these variables affect their biological and therapeutic outcomes. Unfortunately, most of the techniques used to characterize the surface of NPs do not give complete information, and their use is often limited to some classes of ligands, hampering their use for different types of ligands or nanosystems. Increasing the characterization of these NP systems will allow scientists to better compare different systems, reducing the variability in results and allowing them to determine which surface characteristics and conjugation methods are more suitable and effective to yield more efficacious and promising drug delivery systems.

GBM is an incredibly deadly disease, increased by its complexity and difficulty in procuring new effective treatments, even when using advanced NP systems. These studies demonstrate the cutting-edge research to overcome these difficulties. While each small discovery, involving new ligands and improved methodologies that are being researched each day, is critical, a combined effort will be needed to simultaneously cross the BBB express GBM cell-specific targeting, taking advantage of or inhibiting the changes in the TME in order to have a multifunctional system that will be more adept at early-stage diagnosis and more efficacious in late-stage treatments. At the same time, it is pivotal to standardize techniques that can allow for a more detailed characterization of the surface of NPs, in order to have a thorough understanding of the nanosystems being used, with the aim of improving not only therapeutic efficacy, but also reproducibility and production. All the above will require future research to improve the design, formulation, and characterization technologies in order to maximize the curative effect, as well as minimize cancer resistance and recurrence. Altogether, researchers are paving the way to help treat and improve the life expectancy and provide a ray of hope for the future patients that will be diagnosed with this horrible disease.

## Figures and Tables

**Table 1:** Summary of the presented nanosystems for GBM targeting.

NP Type	Targeting Moiety	Target	Key Results	Ref.
Crosslinked peptide	MPC	Acetylcholine transporter	Successful delivery of nimotuzumab in orthotopic glioma xenograft mice	[32]
Liposomes	ApoE derived peptide + chlorotoxin	Lipid transport	Doxorubicin loaded into the liposomes produced reduced viability of GBM U87 cells and did not affect endothelial cells in vitro	[33]
Nano-immune conjugate	Tf Receptor antibody	Tf receptor	PMLA backbone conjugated to Tf Receptor antibody for targeting and CTLA-4 and PD-1 antibodies. The system was able to induce antitumor immune response in GBM mice	[34]
PEGylated liposomes	ITGA2 antibody	ITGA2	Doxorubicin loaded liposomes were able to cross the BBTB but not the healthy BBB. ITGA2 blocked GBM cell migration	[39]
PLGA NPs	M08 antibody	Cell surface vimentin	NPs loaded with paclitaxel showed increased apoptosis in GBM cells compared to healthy astrocytes	[40]
Porous silica NPs	SIWV peptide	Caveolin-mediated transport	Accumulation of NPs in the brain of mice with GBM xenografts, resulting in prolonged survival, with higher GBM selectivity in vitro than in vivo	[42,43]
Lipid-CaP NPs	CTCE9908 peptide	CXCR4	Efficient delivery of siRNA in GBM cultures and GBM mice, resulting in silencing of the PD-1 gene ligand	[45]
PEG–PCL NPs	WGA + FA	Sialic acid + FA receptor	NPs were loaded with different anticancer drugs, and the double-ligand strategy showed improved targeting efficacy compared to the single moieties in vitro	[46]
PEGylated liposomes	VAP + p-HA	GRP78 protein + dopamine receptors	Enhanced BBB crossing and GBM accumulation in spheroids Apoptotic and antiangiogenic effect in orthotopic GBM mice	[47]
PEGylated liposomes	RGD + Lf	Integrin αvβ3 + Lf receptor	Improved BBB crossing and GBM accumulation in spheroids Improved efficacy of docetaxel in vivo compared to nontargeted liposomes	[49]
Polymeric micelles	ST-RAP12 peptide	LRP1 receptor	The peptide improved GBM specificity of paclitaxel-loaded micelles, with increased survival rate and inhibited angiogenesis in vivo	[50]
DSPE–PEG micelles	^D^ATP	Neuropeptide Y receptor Y1	Increased BBB crossing in vitro compared to other known ligands The ATP peptide improved photothermal therapy in vivo	[51]
PEGylated liposomes	RVG15 peptide	Nicotinic acetylcholine receptor	Improved delivery of paclitaxel across the BBB and accumulation in GBM cells in vivo Inhibition of tumor growth and metastases formation	[53]
Liposomes	mnRwr peptide	Integrin αvβ3	Increased penetration in tumor spheroids compared to RGD peptide, and increased accumulation in GBM mice	[54]
PEI-coated silica NPs	T10 peptide	Tf receptor	Induced formation of a Tf corona on the surface of NPs to target the Tf Receptor Efficient BBB crossing and GBM targeting in vivo with prolonged release of doxorubicin	[55]
Albumin NPs	Collagenase	Extracellular matrix	Efficient delivery of gemcitabine in tumor spheroids	[56]
Liposomes	EGF	EGF receptor	Increased delivery of silver NPs loaded into liposomes, specifically to GBM cells in vitro	[57]
Albumin NPs	Scavenger receptor A + SPARC protein	TAMs in TME	Improved ICB therapy with elimination of TAMs from the TME	[58]
Copper–selenium NPs	Biomimetic cell membrane	TME	Shift of TAMs to an M1 phenotype, decreased expression of the PD-1 ligand, and increase in memory T cells	[59]
Albumin NPs	ROS-sensitive linker + PD-1 ligand antibody	ROS in TME	System enclosed in a hydrogel together with iron oxide NPs for combined photodynamic therapy and immunomodulation	[60]
Platinum NPs + dextran NPs	Linkage via pH -sensitive borate ester	Acidic pH in TME	Disassembly of the two NPs improved penetration into GBM and release of loaded sotuletinib to eliminate TAMs	[61]

Abbreviations: BBB—blood-brain barrier; BBTB—blood-brain tumor barrier; CaP—calcium phosphate; EGF—epidermal growth factor; FA—folic acid; GBM—glioblastoma multiforme; ICB—immune checkpoint blockade; Lf—lactoferrin; MPC—2-methacryloyloxyethyl phosphorylcholine; NPs—nanoparticles; PD-1—programmed cell death 1- (ligand, antibody, etc); PEG-PCL—poly(ethylene glycol)–poly(ε-caprolactone); PEI—polyethyleneimine; p-HA—p-hydroxybenzoic acid; PMLA—poly(β-L-malic acid); ROS—reactive oxygen species; TAMs—tumor-associated macrophages; Tf—transferrin; TME—tumor microenvironment; VEGF—vascular endothelial growth factor; WGA—wheat germ agglutinin.

## Data Availability

Not applicable.
